# *In Situ* Peptide-MHC-II Tetramer Staining of Antigen-Specific CD4^+^ T Cells in Tissues

**DOI:** 10.1371/journal.pone.0128862

**Published:** 2015-06-11

**Authors:** Thamotharampillai Dileepan, Hyeon O. Kim, P. Patrick Cleary, Pamela J. Skinner

**Affiliations:** 1 Department of Microbiology, University of Minnesota, Minneapolis, MN, United States of America; 2 Department of Veterinary and Biomedical Sciences, University of Minnesota, Saint Paul, MN, United States of America; Johns Hopkins University, UNITED STATES

## Abstract

The invention of peptide-MHC-tetramer technology to label antigen-specific T cells has led to an enhanced understanding of T lymphocyte biology. Here we describe the development of an *in situ* pMHC-II tetramer staining method to visualize antigen-specific CD4^+^ T cells in tissues. This method complements other methods developed that similarly use MHC class II reagents to stain antigen-specific CD4^+^ T cells *in situ*. In this study, we used group A streptococcus (GAS) expressing a surrogate peptide (2W) to inoculate C57BL/6 mice, and used fresh nasal-associated lymphoid tissues (NALT) in optimizing the *in situ* staining of 2W:I-A^b^ specific CD4^+^ T cells. The results showed 2W:I-A^b^ tetramer-binding CD4^+^ T cells in GAS-2W but not GAS infected mice. This method holds promise to be broadly applicable to study the localization, abundance, and phenotype of antigen-specific CD4^+^ T cells in undisrupted tissues.

## Introduction

The development of peptide-MHC-II (pMHC-II) tetramer staining has revolutionized our ability to study antigen specific CD4^+^ T cells [1–3]. Although *in situ* staining of antigen specific CD8^+^ T cells with pMHC-I tetramers has been well characterized and permits visualization and characterization of antigen-specific CD8^+^ T cells relative to other cell types in stained undisrupted tissue sections [4–6], similar staining of antigen specific CD4^+^ T cells *in situ* with pMHC-II tetramers has been later in coming. Several groups including ours have now developed methods using MHC class II reagents to visualize antigen-specific CD4^+^ T cells in tissues with their spatial relationship to other cells intact. Li et al., used HLA tetramers on frozen and fixed lymph node and lung tissue sections to label CD4 T cells specific for *Mycobacterium tuberculosis* [7]. Bischof et al., used mouse class II I-A^s^ tetramers to label self-reactive CD4 T cells in fresh PBS perfused lymph node and central nervous tissues from experimental autoimmune encephalomyelitis (EAE) mice [8]. Similarly Massilamany et al., labeled self-reactive CD4 T cells in fresh brain tissue sections from EAE mice using mouse using MHC class II I-A^s^ dextramers, and also used class II I-A^k^ dextramers to label self-reactive CD4 T cells in fresh heart tissue sections from experimental autoimmune myocarditis mice [9,10]. Here we describe the successful development of an additional *in situ* staining technique using pMHC-II tetramers to visualize antigen specific CD4^+^ T cells in tissues with their spatial relationship to other cells intact.

Previously, we used T cell receptor (TCR) transgenic mice to optimize *in situ* detection of antigen-specific CD8^+^ T cells [4], where the vast majority of T cells are identical. In this study we used a more realistic system where we targeted endogenous antigen-specific T cells in mice that were inoculated with a bacterial pathogen namely, group A *Streptococcus pyogenes* (GAS). We used a recombinant GAS strain (GAS-2W) that expresses an immunogenic peptide (EAWGALANWAVDSA) called 2W [11] fused to the M1 protein on its surface to intranasally inoculate mice. After multiple inoculations, nasal-associated lymphoid tissue (NALT) and spleens were used for IST. Our strategy involved making 2W:I-A^b^ tetramers with ExtraAvidin-FITC, and then using FITC as an epitope to amplify the tetramer signal in IST, and performing IST with fresh tissue sections. We performed parallel flow cytometry analysis of NALT from littermates to validate the IST. We used NALT and spleen tissues from C57BL/6 mice inoculated with wild-type GAS missing the 2W epitope as a negative control. In this report, we describe an *in situ* MHC-class II tetramer staining technique that should be generally applicable to visualizing antigen-specific CD4 T cells in tissues.

## Materials and Methods

### Generation of peptide-MHC-II (2W:I-A^b^) tetramers

2W:I-A^b^ tetramers were designed and produced as previously described with slight modifications [1]. 2W:I-A^b^ molecules were expressed in Drosophila melanogaster S2 cells using the Drosophila Expression System kit (Invitrogen). Briefly, pRMHa-3 vectors containing the alpha and beta chains of I-Ab under the control of the metallothionein promoter were used to generate monomers. Sequences encoding 2W peptide (EAWGALANWAVDSA) was fused to the N terminus of the beta chain via a flexible polyglycine linker (GGGGTSGGGSGGS). C-terminal fusions of acidic and basic leucine zipper domains forced heterodimerization. A 6 x His epitope tag on the beta chain and a single biotinylation on the alpha chain facilitated purification and tetramerization. Drosophila S2 cells were cotransfected with plasmids encoding the I-Ab alpha chain, beta chain, BirA ligase and a blasticidin resistance gene at a ratio of 9:9:9:1, using calcium phosphate transfection kit (44–0052, Invitrogen). Transfected cells were selected in blasticidin-containing serum-free media at 28C, scaled up to one liter cultures (~107 cells/ml) in spinner flasks maintained at 120 rpm and induced by the addition of 0.8 mM copper sulfate and supplemented with D-biotin (2 μg/ml final conc.) for *in vivo* bitinylation. Soluble 2W:I-A^b^ monomers were purified from the cell culture supernatant via nickel affinity chromatography, followed by an additional purification on a Pierce Monomeric Avidin UltraLink Resin (Thermo Scientific). The biotinylated monomers were eluted with free 2 mM D-biotin and washed using Amicon Ultra 30kD concentrating filters (Merck Millipore Ltd.) to remove excess free biotin and concentrate the monomers. The resulting product was then used to generate tetramers with ExtrAvidin−FITC (Sigma).

### Bacterial strains and growth

We grew streptococci in Todd-Hewitt broth supplemented with 2% neopeptone (THB-Neo) or on solid media containing Difco blood agar base and sheep blood at 37°C in 5% CO_2_. We purchased all growth media from Difco Laboratories, Detroit, MI. Strain 90–226 (serotype M1) was originally isolated from the blood of a septic patient [12]. Generation of the recombinant GAS strain that expresses the 2W epitope in M protein designated Streptococcus pyogenes 90–226 emm1.0::2W (GAS-2W) has been described previously [11].

### Inoculation of mice

We purchased six weeks old female C57BL/6 mice from the National Cancer Institute (NCI, Frederick, MD). We anesthetized mice with isoflurane/oxygen mixture and inoculated intranasally with GAS-2W by placing 2 X 10^8^ CFU in a total volume of 15 μl PBS (7.5 μl /nostril) with a 10 μl pipette. We inoculated control mice with same dose of wild-type GAS (GAS-WT). Inoculations were repeated at weekly intervals for three weeks. Mice were housed under specific pathogen-free conditions at Research Animal Resources (RAR) facilities of University of Minnesota. Mice inoculated with GAS were housed in biosafety level 2 facilities of the RAR. All experiments were conducted according to institutional guidelines using protocols approved by the Institutional Animal Care and Use Committee (IACUC) of the University of Minnesota.

### Ethics Statement

The University of Minnesota has an approved Animal Welfare Assurance #A3456-01 on file with the NIH Office of Laboratory Animal Welfare and complies with the USDA Animal Welfare Act Regulations, and the Public Health Service Policy on Humane Care and Use of Laboratory Animals. All animal studies at the University of Minnesota were carried out in strict accordance with the recommendations in the Guide for the Care and Use of Laboratory Animals of the National Institutes of Health. The Academic Health Center is fully accredited by the Association for the Assessment and Accreditation of Laboratory Animal Care, International. The University of Minnesota Institutional Animals Care and Use Committee (IACUC) approved our animal use protocol.

### Detection of 2W:I-A^b^-specific CD4^+^ T cells in situ

We used fresh NALT and spleen from six GAS-2W and five control GAS infected mice for these studies. For *in situ* tetramer staining, we cut fresh tissues into approximately 0.5-cm pieces and embedded in 4% low-melt agarose. Tissue blocks were placed in a vibratome bath containing 0 to 4°C PBS with 100 μg/ml heparin (PBS-H), and 200 μm thick sections were generated. To work out staining conditions, we tested several tetramer concentrations, incubation times, and temperatures as shown in Table 1. Two staining conditions, one with and one without tyramide signal amplification (TSA), worked best and are described as follows. Sections were incubated at 4°C overnight followed by 1 hour incubation at room temperature with 2 nM FITC-labeled 2W:I-A^b^ tetramers in 1 ml PBS-H with 2% normal goat serum (NGS). Sections were then washed with chilled PBS-H and fixed with fresh 4% paraformaldehyde for 2 hours at room temperature. Sections were again washed with PBS-H, incubated with rabbit anti-FITC antibodies (AbD Serotec) diluted 110,000 in PBS-H with 2% NGS at 4°C on a rocking platform overnight. Sections were washed three times with PBS-H for at least 20 min each wash and then incubated with Cy3-conjugated donkey or goat anti-rabbit antibodies diluted 12000 (Jackson ImmunoResearch), in PBS-H with 2% NGS, for 1 to 3 days at 4° on a rocking platform in the dark (wrapped plated in tin foil). No difference in staining was noted between 1 and 3 day final incubations. Finally, sections were washed three times for at least 20 min each wash in PBS-H, post-fixed with 4% paraformaldehyde for 1 hour, and then mounted on slides with warmed glycerol gelatin (Sigma) containing 4 mg/ml propyl gallate (Fluka). For TSA amplification, we processed sections as described above through the step of incubating tissues with anti-FITC antibodies and washing. Then endogenous peroxidases in sections were blocked using 3% H_2_O_2_ for 1 hour at RT following by washing in PBS-H, sections were then incubated with goat anti-rabbit HRP (Jackson ImmunoResearch, 1:2000) in PBS-H with 2% NGS, for 1 to 3 days and washed in PBS-H, then incubated with the TSA Plus Cyanine 3 reagent (PerkinElmer) for 1hr at RT following by washing in PBS-H, and post-fixed and mounted as described above. During tetramer staining, sections were also counterstained with antibodies including rat anti-mouse CD4 (eBioscience, clone RM4-5, clone GK1.5) or rat-anti-mouse CD3 (AbD Serotec, clone CD3-12) antibodies diluted 1200, followed by fluorophore-conjugated donkey or goat anti-rat antibodies diluted 12000 (Jackson ImmunoResearch) to label T cells, and goat-anti-mouse IgG (Invitrogen) diluted 1:2000 to label B cells. To rule out non-specific binding of antibodies, in a subset of sections, primary antibodies were excluded and tissue sections were stained with only fluorophore-conjugated secondary antibodies. We analyzed stained sections using an Olympus FV1000 confocal microscope. For each section, several fields were collected at multiple z-depths using a 20X objective and stitched together to form 3-demensional montage images. Images were analyzed using Olympus FluoView Viewer 2.0 and Adobe Photoshop 7.01 software. For cell quantification, at least 25 to 105 (average 54) tetramer-binding cells were evaluated per sample for each animal.

**Table 1:** p-MHC-II in situ tetramer staining conditions and results.

Incubation time and temperature	Tetramer concentration	TSA amplification	Tetramer staining intensity over background	Background levels
1 HR RT	2nM		none	+
	20nM		none	+++
3 HR RT	2nM		none	+
	20nM		none	+++
**4°C O/N plus 1hr RT**	**2nM**		**++**	**+**
	4nM		++	++
**4°C O/N plus 1hr RT**	0.1nM	one round	none	+
	**2nM**	**one round**	**+++**	**+++**
4°C O/N plus 1hr RT	0.1nM	two rounds	+	++++
	1nM	two rounds	+	++++
	2nM	two rounds	+	++++

### Flow cytometry

2W:I-A^b^ specific CD4^+^ T cells were analyzed as described previously [1–3]. Briefly, mice were euthanized and NALT and spleen were harvested separately in cEHAA medium. Single cell suspensions were prepared in 200 μl of Fc block (supernatant from 2.4G2 hybridoma cells grown in serum-free media with 2% mouse serum, 2% rat serum, 0.1% sodium azide) from each tissue. ExtrAvidin−FITC conjugated tetramer was added at a final concentration of 10 nM and the cells were incubated at room temperature for 1 hr. Following tetramer staining all samples underwent surface staining on ice with APC-conjugated anti-CD4 (RM4-5); PerCP-conjugated anti-CD3ϵ (145-2C11); Alexa Fluor 700-conjugated anti-CD44 (IM7); as well as Pacific Blue-conjugated dump channel antibodies specific for B220 (RA3-6B2), CD11b (MI-70), CD11c (N418). We used a Becton Dickinson LSR-II flow cytometer to collect and analyze events that have the light scatter properties of lymphocytes. Acquisition was performed using FACSDiva software (BD) and analysis was done using FlowJo (Tree Star, Ashland, OR).

## Results and Discussion

To develop a method using peptide-MHC class II tetramers to identify antigen-specific CD4^+^ T cells *in situ*, we chose to use an intranasal mouse infection model using a recombinant Group A Streptococci expressing a model epitope, 2W, fused to the M1 protein (GAS-2W) [11]. Intranasal GAS infections induce a strong antigen specific Th17 response in nasal-associated lymphoid tissue (NALT) of mice and multiple infections increase the number of these cells in NALT [11,13]. C57BL/6 mice were inoculated three times intranasally at weekly intervals with GAS-2W. As a negative control, a group of mice was similarly inoculated with wild-type GAS that does not express the 2W epitope (GAS-WT).

**Fig 1:**
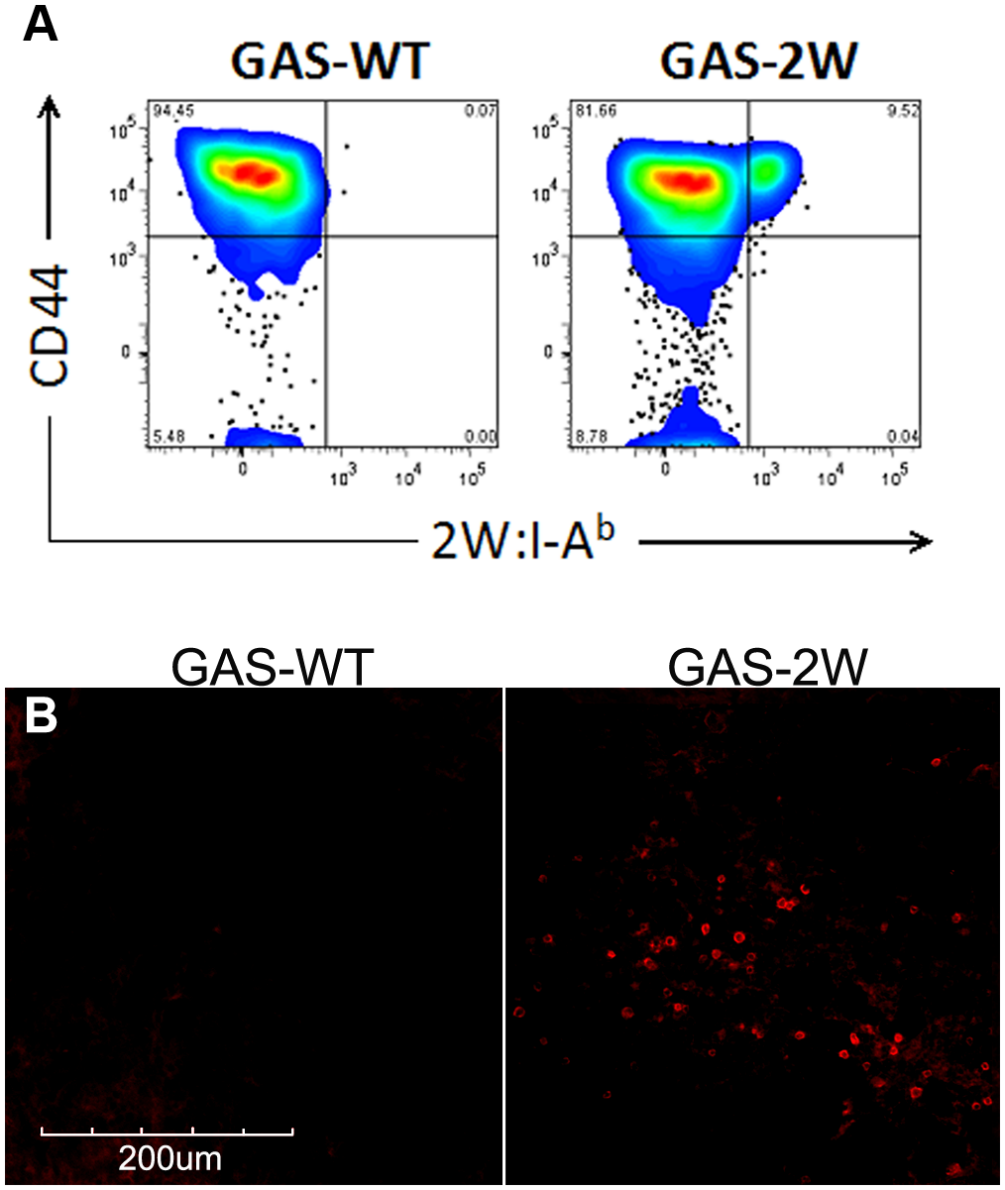
2W:I-A^b^ tetramer staining via flow cytometry and *in situ* staining. 2W:I-A^b^-ExAv-FITC tetramer identified an antigen-specific CD4^+^ T cell population that had expanded in response to intranasal GAS-2W infection. A) Flow cytometric analysis of 2W:I-A^b^ tetramer+ cells from disaggregated NALT showed populations of tetramer+CD4+CD44+ cells in GAS-2W but not GAS mice. Cells were gated on total CD4^+^ cells. *B*) 2W:I-A^b^ tetramer *in situ* staining of NALT from littermates showed tetramer+ cells in GAS-2W infected mice that were above background levels of staining detected in similar regions of NALT in the negative control GAS infected mice. For these tissue sections, *in situ* tetramer staining was done with TSA amplification.

Three days after the last infection mice were sacrificed and half of each group was used for *in situ* staining and half used in parallel flow cytometry analysis. Flow cytometry of single cell suspensions from NALT and spleen stained with 2W:I-A^b^-ExAv-FITC tetramers identified an antigen-specific CD4^+^ T cell population that expanded in response to intranasal GAS-2W infection and stained positively with antibodies directed against the CD44, a marker for effector and memory T cells (Fig 1A, right panel). These cells were absent in NALT from the control mice that were infected with the wild-type GAS that does not express the 2W epitope (Fig 1A, left panel). We did not detect any tetramer^+^ cells in the spleen via flow cytometry without magnetic bead enrichment (data not shown). This flow cytometry analysis confirmed that the newly generated 2W:I-A^b^-ExAv-FITC tetramer would identify antigen-specific CD4 T cells in NALT, the site of GAS infection, where considerable numbers of these cells accumulate, and that we could readily identify these cells without magnetic bead enrichment. This relatively high abundance of target cells made NALT an ideal tissue to optimize pMHC-II staining.

Because in previous studies we found class I tetramer staining *in situ* worked best with fresh, unfixed, tissue sections [4–6], we used fresh tissues in this study. We tested multiple tetramer concentrations, incubation times, and temperatures as presented in Table 1. The two staining conditions that worked the best to detect tetramer-stained cells above background are highlighted in yellow in Table 1 and described here. For this process, we embedded NALT and spleen tissues isolated from GAS-2W and wild-type GAS infected mice in 4% low-melt agarose and sectioned with a vibratome to generate 200-μM thick sections followed by staining with 2W:I-A^b^-ExAv-FITC tetramers overnight at 4° plus 1 hour at 37°. To detect bound tetramers, we amplified the signal using anti-FITC antibodies, and then used either Cy3-conjugated antibodies directed against the FITC antibodies, or HRP-conjugated antibodies directed against the FITC antibodies followed by TSA amplification. We detected 2W:I-A^b^ tetramer+ cells in NALT of GAS-2W that were above background levels of staining detected in negative control mice infected with wild-type GAS, and were of the expected size and shape of T cells (Fig 1B). No tetramer+ cells were detected above background *in situ* in spleen tissues (data not shown). Although tetramer staining conditions with and without TSA amplification were established that resulted in detection of tetramer-binding cells above background, we found that TSA amplification showed substantially brighter tetramer-binding cells as well as increased overall non-specific background staining compared to no TSA amplification (Table 1 and S1 Fig).

**Fig 2:**
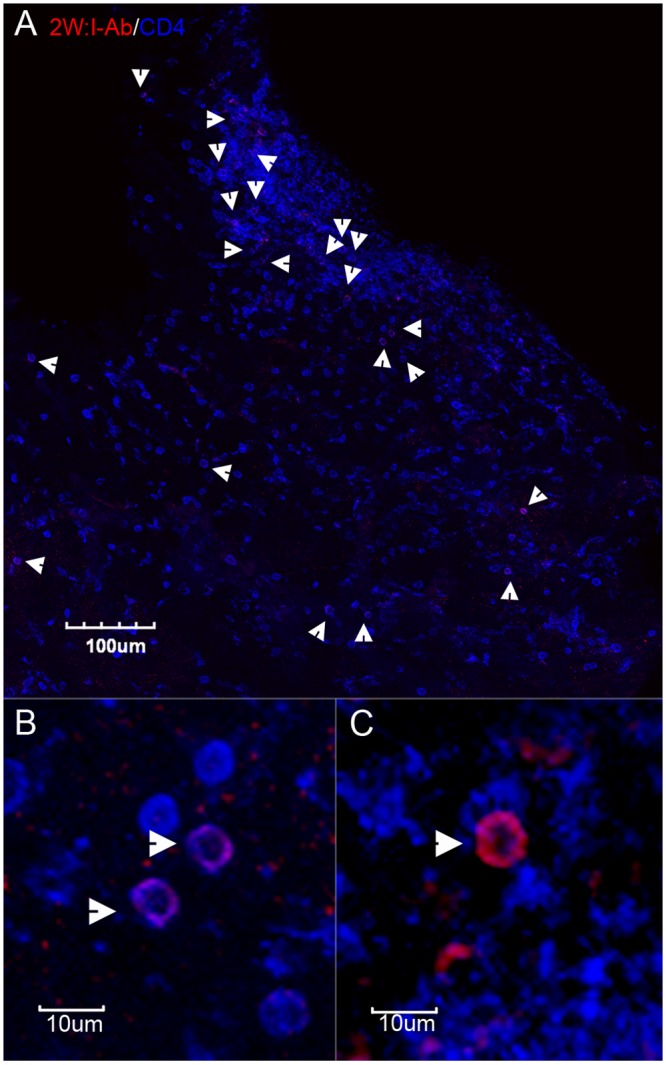
Populations of 2W:I-A^b^ tetramer+ cells detected *in situ* in NALT co-stained with CD4 antibodies. (*A*) Representative image of a NALT section stained with 2W:I-A^b^ tetramers with no TSA amplification (red) and CD4 antibodies (blue). 2W:I-A^b^ tetramer^+^ cells that were co-labeled with CD4 are indicated by arrowheads. In this field, all of the tetramer^+^ cells are CD4^+^. For this image, several confocal z-scan fields were collected using a 20X objective and reconstructed as a montage. (*B*) Shows two 2W:I-A^b^ tetramer-binding CD4^+^ T cells (indicated by arrowheads). (*C*) Shows a 2W:I-A^b^ tetramer-binding cell (red) from another field that is not co-stained with CD4 antibodies. (*B*) and (*C*) are confocal z-scans collected using the same parameters with a 60X objective.

**Fig 3:**
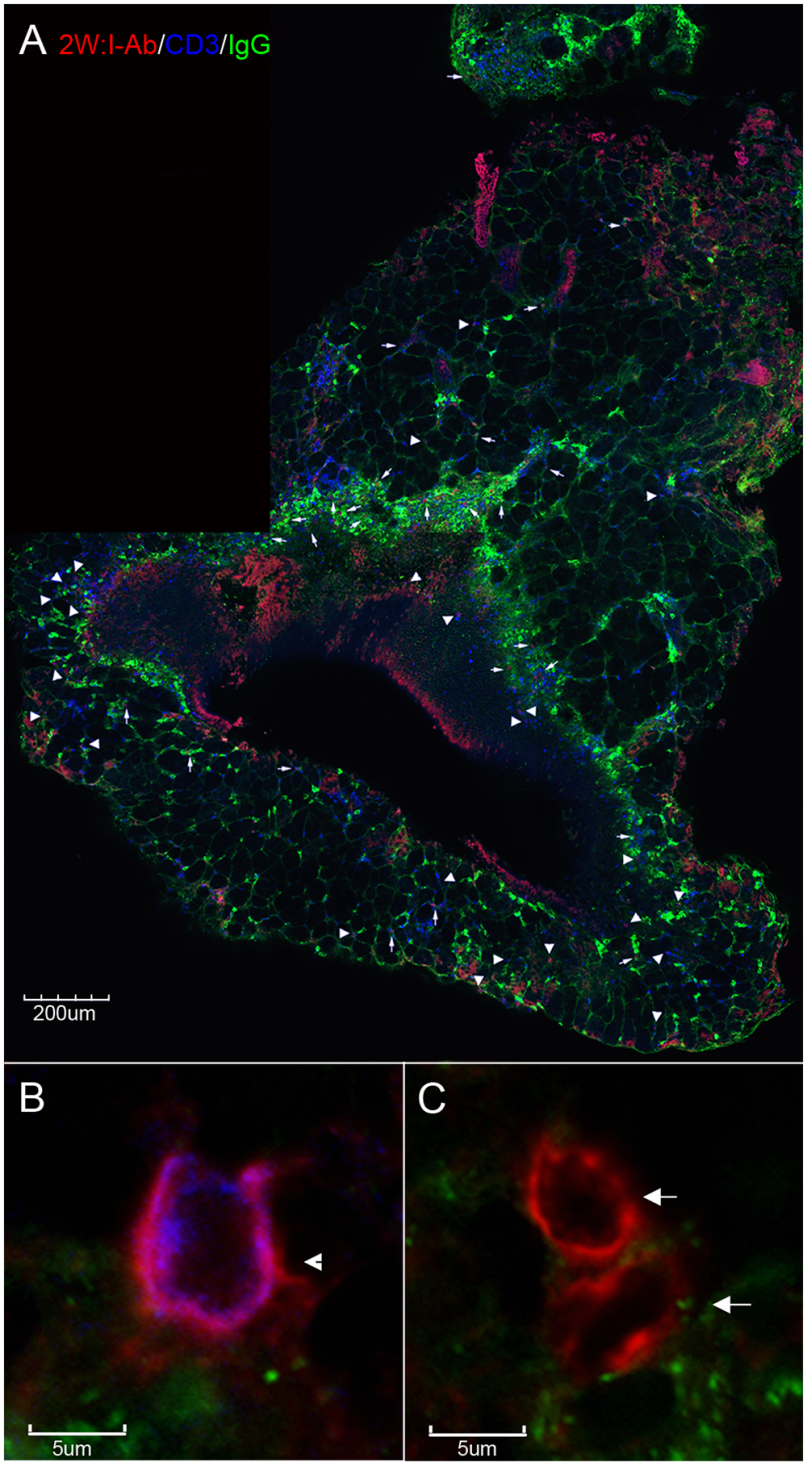
Population of 2W:I-A^b^ tetramer+ cells detected *in situ* in NALT co-stained with CD3 but not IgG antibodies. (*A*) Representative image of a whole NALT section from a GAS-2W infected mouse stained with 2W:I-A^b^ tetramers (red) with one round of TSA amplification, CD3 antibodies (blue), and IgG antibodies (green). For this image, several confocal z-scan fields were collected using a 20X objective and reconstructed as a montage. The enlargement in (*B*) shows a 2W:I-A^b^ tetramer-binding T cell that is co-stained with CD3 antibodies but not IgG antibodies. The enlargement in (*C*) shows 2W:I-A^b^ tetramer-binding cells that are not co-stained with CD3 or IgG antibodies. (*B*) and (*C*) are confocal z-scans collected using the same parameters with a 60X objective and zoom of 3. 2W:I-A^b^ tetramer^+^CD3^+^IgG^-^ cells are indicated with arrowheads, and 2W:I-A^b^ tetramer^+^CD3^-^IgG^-^ cells are indicated with arrows.

In order to differentiate tetramer+ antigen-specific CD4^+^ T cells from other cells that bind tetramers, we co-stained tissue sections with anti-CD4 or anti-CD3 antibodies to label T cells and anti-IgG antibodies to label IgG expressing B cells and plasma cells. 2W:I-A^b^ tetramer staining combined with anti-CD4 staining revealed populations of 2W:I-Ab tetramer^+^ cells co-stained with anti-CD4 antibodies distributed amongst other CD4+ cells in NALT tissues from GAS-2W infected mice (Fig 2). In addition, many non-CD4 cells were also 2W:I-A^b^ tetramer^+^ but these were easily distinguishable from antigen-specific tetramer^+^CD4^+^ T cells through CD4 co-staining (Fig 2 panels B and C). Similar 2W:I-A^b^ tetramer^+^ CD4^-^ cells were also detected in negative control animals (S1 Fig). We have historically found that CD3 antibody staining is generally superior in quality and more reliable than CD4 staining of T cells in tissue sections. Given this, we also stained sections with CD3 antibodies. Since class II tetramers do not bind CD8 T cells [1] we reasoned that CD3 antibodies are a good alternative to CD4 antibodies to identify antigen-specific tetramer^+^ CD4^+^ T cells. Fig 3A shows an image of a whole NALT section stained with 2W:I-A^b^ tetramers and counterstained with anti-CD3 and anti-IgG antibodies. Fig 3B shows a representative 2W:I-A^b^-binding T cell that was co-stained CD3 antibodies. Fig 3C shows representative tetramer-binding cells that were not co-stained with CD3.

**Fig 4:**
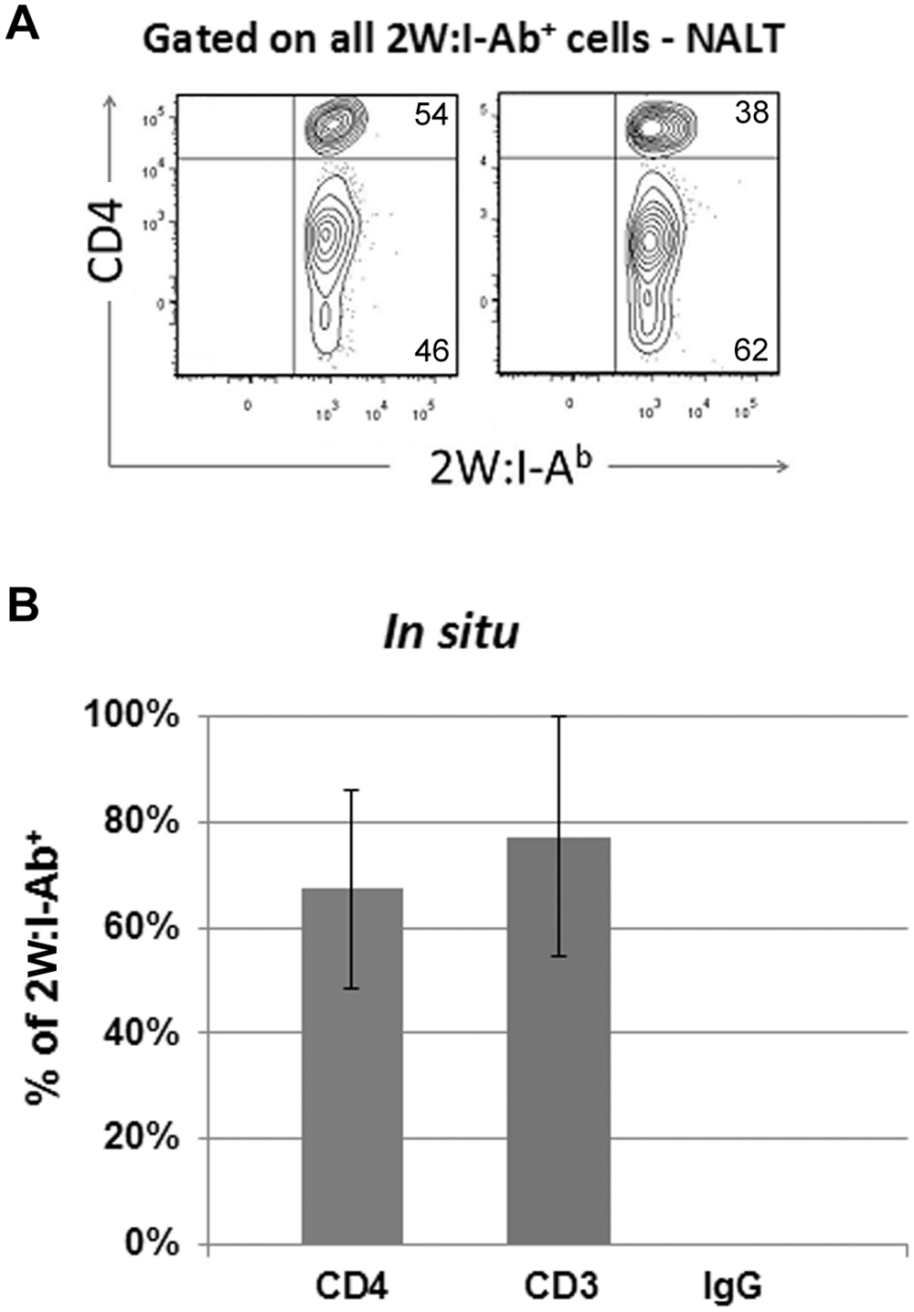
Percentage of 2W:I-A^b^ tetramer binding cells that counterstained with CD3 and CD4 antibodies. (*A*) Flow cytometry plots from the NALT of two GAS-2W infected mice showing the percentage of 2W:I-A^b^ tetramer-binding cells that were co-stained with CD4 antibodies. Cells were gated on all cells stained with 2W:I-A^b^-ExtrAvidin−FITC tetramers. (*B*) Shows the average percentage of 2W:I-A^b^ binding cells from four mice that were co-stained with CD4, CD3, and IgG antibodies *in situ*, with standard deviations indicated.

We next determined the percentage of tetramer-binding cells that co-stained with CD4, CD3, and IgG antibodies in the NALT from four mice stained *in situ* and in two littermate mice co-stained with CD4 antibodies via flow cytometry. In the two mice analyzed via flow cytometry, 54% and 38% of 2W:I-A^b^ tetramer^+^ cells were co-stained with CD4 antibodies (Fig 4A). In the four animals analyzed *in situ*, an average of 67% (range 50–90%) of 2W:I-A^b^ tetramer+ cells were co-stained with CD4 antibodies; an average of 83% (range 53–100%) co-stained with CD3 antibodies; and none of the tetramer+ cells co-stained with IgG antibodies (Fig 4B). Thus, there was variability amongst individual animals with regard to the percent of tetramer-binding cells that co-stained with CD4 and CD3 antibodies, but in all animals, at least half to almost all tetramer-binding cells were co-stained with these T cell markers *in situ*.

These results also demonstrate that the 2W:I-A^b^ tetramer+ cells identified *in situ* that did not co-stain with CD3 antibodies are not IgG+ B cells. Although not IgG positive, at least some of the tetramer^+^CD3^-^ cells detected may be IgM or another isotype of antibody expressing B cells. Naive populations of B cells specific to the fluorophores phycoerythrin (PE) and allophycoerythrin (APC) have been detected in B6 mice [14]. Given this, there may similarly be naive populations of B cells specific for the fluorescein (FITC) fluorophore used to make the 2W:I-A^b^ tetramers used in this study.

In this study, we used tissues from wild-type GAS infected animals that did not express the CTL epitope for which our class II tetramers bound. It is important to note an alternative negative control that can be used for *in situ* class II reagent staining, when tissues from animals that do not express the CTL epitope are unavailable, or less feasible. A very good alternative negative control is tetramers or dextramers with MHC-class II molecules loaded with irrelevant peptides [8,9].

In summary, we have developed a technique using pMHC-II tetramers to stain antigen-specific CD4^+^ T cells *in situ*. We successfully used pMHC-II tetramers to detect 2W:I-A^b^-specific CD4^+^ T cells in NALT tissue sections from GAS-2W infected C57BL/6 mice. In addition, we illustrate the necessity of co-staining with anti-CD4 or anti-CD3 antibodies in order to distinguish tetramer-binding CD4^+^ T cells from the other cell types that the tetramers bind. This study complements other recent studies describing methods using pMHC-II tetramers and dextramers to label antigen-specific CD4 T cells *in situ* [7–10]. These methodologies hold promise to be broadly applicable valuable tools for future studies of the localization, abundance, and phenotype of potentially any antigen-specific CD4^+^ T cells in any tissue from which fresh sections can be generated and for which pMHC-II tetramers are available.

## Supporting Information

S1 Fig Comparison of 2W:I-A^b^ tetramer staining with and without TSA amplification in GAS-2W infected and negative control GAS infected mice. Representative images of NALT sections stained with 2nM 2W:I-A^b^ tetramers without TSA amplification (A and C) and with TSA amplification (B and D), from a GAS-2W infected mouse (A and B) and a negative control GAS infected mouse (C and D). For these confocal z-scans, the red intensity was increased equally in all images using the curves tool in Photoshop. The insert in (C) includes the CD4 counterstaining (blue), and shows that the tetramer+ cell is not CD4+. Scale bars are 20 μm.
(TIF)
