# Supplementary figures and images for: In Situ Peptide-MHC-II Tetramer Staining of Antigen-Specific CD4+ T Cells in Tissues
Source: PLoS One. 2015 Jun 11;10(6):e0128862. doi: 10.1371/journal.pone.0128862 (PMC4465905; doi:10.1371/journal.pone.0128862)

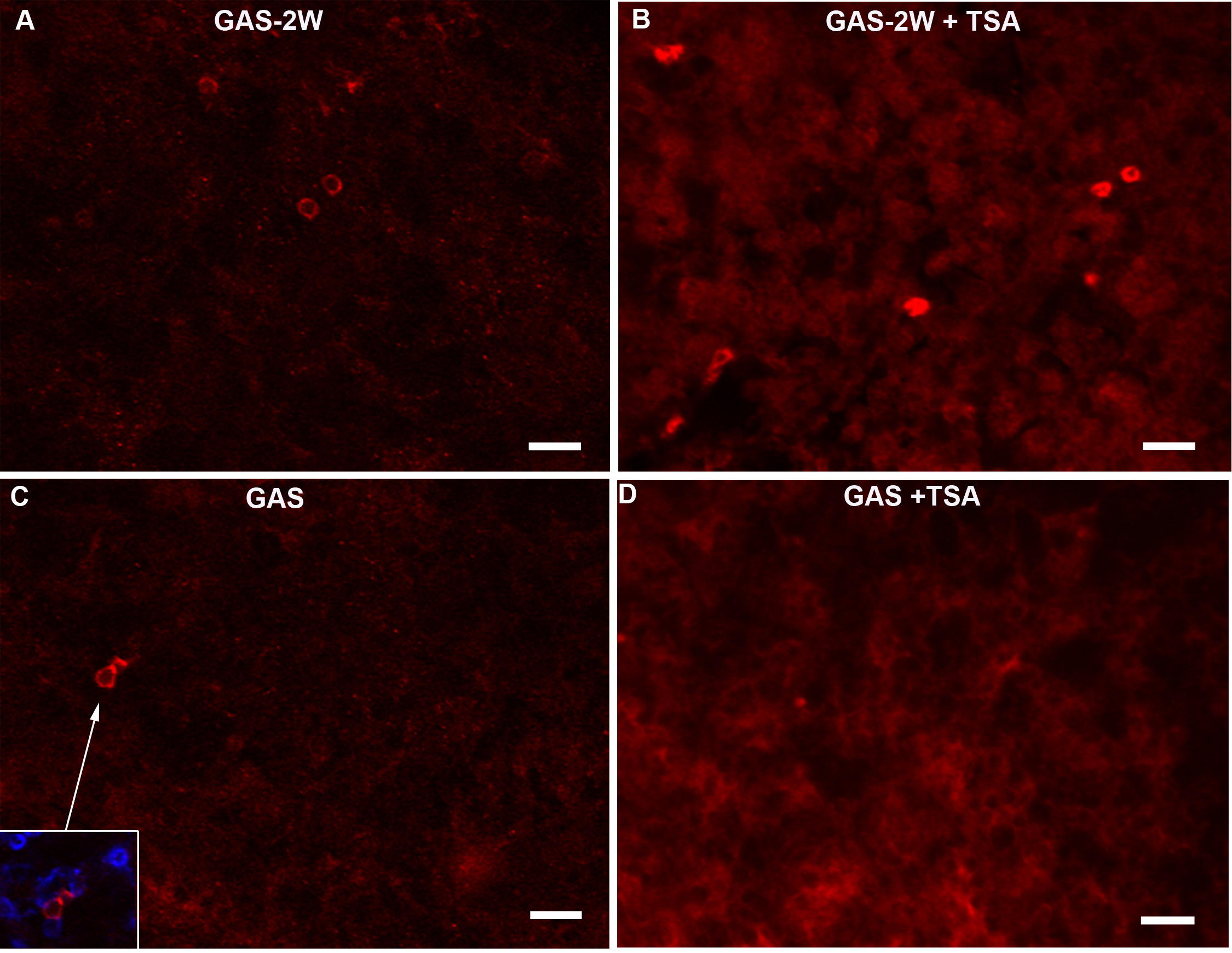

Supplement: S1 Fig — Representative images of NALT sections stained with 2nM 2W:I-Ab tetramers without TSA amplification (A and C) and with TSA amplification (B and D), from a GAS-2W infected mouse (A and B) and a negative control GAS infected mouse (C and D). For these confocal z-scans, the red intensity was increased equally in all images using the curves tool in Photoshop. The insert in (C) includes the CD4 counterstaining (blue), and shows that the tetramer+ cell is not CD4+. Scale bars are 20 μm. (TIF) [file pone.0128862.s001.tif]
